# Self-other differences in the perceived authenticity of attitudes expressed toward social groups

**DOI:** 10.3389/fpsyg.2024.1467396

**Published:** 2024-12-16

**Authors:** André Mata, André Vaz

**Affiliations:** CICPSI, Faculdade de Psicologia, Universidade de Lisboa, Lisbon, Portugal

**Keywords:** better-than-average effect, pluralistic ignorance, perceived authenticity, true self, self-other differences, dual-process models

## Abstract

We assessed the perceived authenticity of attitudes expressed toward several social groups as a function of whether those attitudes were expressed by the self or by other people, and whether those expressions were automatic (without time to ponder) or controlled (without time constraints). Participants considered their controlled responses more authentic than their automatic responses. However, the same did not happen when considering others’ attitudes. Implications for social perception are discussed.

## Introduction

Most people consider themselves to be better than the average other person (e.g., Alicke et al., 1995; Epley and Dunning, 2000). These comparative biases extend to prejudice, where people assume that others are generally more endorsing of prejudiced attitudes than they are (Bell et al., 2019; Fields and Schuman, 1976; Howell and Ratliff, 2017; Kotzur et al., 2020; O'Gorman and Garry, 1976; Saucier, 2002). In part, this pluralistic ignorance (Prentice and Miller, 1993, 1996) stems from people’s tendency to reject evidence suggesting they are prejudiced: They are defensive toward information that threatens their non-prejudiced self-image (Howell and Ratliff, 2017), questioning its relevance and validity (Howell et al., 2017; Howell et al., 2015; Mendonça et al., 2019; Vitriol and Moskowitz, 2021), or outright avoiding it (Howell et al., 2013).

One possibility for why people act defensively toward such feedback is that the notion that they are prejudiced is incongruent with how they perceive their authentic selves. Indeed, the better-than-average effect extends to people’s notions of authentic selves: Generally, people believe their true or authentic self is better than the average true self of others (Zhang and Alicke, 2021). More generally, people perceive authenticity as socially desirable and strive to show it (Hart et al., 2020) and interpret self-relevant information in ways congruent with a positive authentic self (Seto and Schlegel, 2018; Steimer et al., 2019). Thus, information that may call into question the character of one’s authentic self is felt as a threat.

### Authenticity

Even though there is disagreement regarding the definition of authenticity (Lehman et al., 2019b; Newman, 2016), broadly defined, authenticity refers to “that which is perceived to be “real” or “genuine” or “true”” (Lehman et al., 2019a, p. 19). Specifically, in the present study, we will consider authenticity as the true expression of one’s values and beliefs (Dutton, 2003; i.e., what Lehman et al., 2019b, refer to as consistency). Being authentic means that “one acts in accord with the true self, expressing oneself in ways that are consistent with inner thoughts and feelings” (Harter, 2002, p. 382).

Authenticity has gained considerable attention in both fundamental and applied research recently (Lehman et al., 2019b; Luthans and Avolio, 2003), and it has proved relevant for both self-perception and wellbeing (Rivera et al., 2019; Schlegel et al., 2009; Schlegel and Hicks, 2011), as well as the perception of other people (e.g., Stiers et al., 2021). Moreover, it has been at the center of research on how to tackle various personal and societal challenges in these turbulent times (e.g., Luthans and Avolio, 2003; Seligman, 2004).

Here, we investigate the perception of the authenticity of attitudes that people express toward social groups. Specifically, we compare the perceptions people have of the attitudes that they express versus those expressed by other people (i.e., self-other differences) in order to test a motivated reasoning account (see the rationale on defensive processing below). We also compare the perceived authenticity of automatic and controlled attitudinal responses and outline hypotheses on how these might differ (based on dual-process accounts), as we shall explain now.

### Dual-process models of prejudice and self-other differences

Dual-process models of prejudice (e.g., Bodenhausen et al., 2009; Crandall and Eshleman, 2003; Devine, 1989; Govorun and Payne, 2006; Son Hing et al., 2002) suggest that people can have both automatic, intuitive attitudes toward others, as well as controlled, deliberative ones, and these need not coincide. When automatic attitudes toward certain groups are more negative than controlled ones, defensiveness to feedback increases (Howell et al., 2017; Mendonça et al., 2019). This might be either because people are aware of their negative automatic attitudes (Goedderz and Hahn, 2022; Zahra et al., 2022) but wish to reject their negative implications, or because they perceive the feedback as incongruent with their positive controlled attitudes and favor their controlled responses as more authentic. In either case, negative automatic attitudes toward stigmatized groups are likely to be seen as less authentic for the self. For other people, however, this need not be the case. Information suggesting that others are prejudiced is not self-threatening or incongruent with people’s general impressions. Moreover, when attitudinal conflict is high (i.e., when there is a large difference between controlled and automatic attitudes), people might be aware of their automatic responses and expect that other people might give those responses (Mata, 2019, 2020; Mata et al., 2013a, b; Simão and Mata, 2023). Therefore, others’ negative automatic attitudes might be seen as more authentic. Indeed, Mendonça et al. (2019) showed how a favorable IAT result is perceived as more valid for the self than it is for others, and this tendency is reversed for unfavorable results. More recently, Garrison et al. (2022) showed that people regard self-controlled actions as more authentic for the self, whereas impulsive actions are seen as more authentic for others. The same might hold for attitudes toward social groups. At the same time, if there is some awareness of an attitudinal conflict (i.e., if one realizes one’s controlled responses differ from one’s automatic response impulses), one might question the authenticity of those responses, even for the self.

If the threat to one’s self-concept drives defensiveness in the form of perceiving automatic prejudice as less authentic, particularly when it is incongruent with controlled attitudes, and when one is aware of this conflict, then reducing the difference between automatic and controlled attitudes should decrease this tendency. Crandall and Eshleman (2003) proposed a justification-suppression model, suggesting that prejudice is suppressed when negative, automatic attitudes go against beliefs and social norms. Decreasing the undesirableness of negative attitudes should, therefore, allow prejudice to manifest in controlled responses. Such is the case for social groups for whom prejudice is prescribed: Groups toward whom harboring prejudice is socially acceptable (Crandall et al., 2002). For those groups, attitudinal conflict should decrease, as both automatic and controlled attitudes should be negative and regarded as authentic.

### Overview

The present paper adapts the two-response paradigm (Thompson et al., 2011) to assess participants’ intuitive/automatic (fast) and deliberative/controlled (slow) responses regarding groups for whom prejudice is either socially prescribed or socially proscribed. We then measure the perceived authenticity of fast and slow attitudes toward these groups. We manipulate whether those attitudinal responses are presented as coming from the self or other people.

We tested the following hypotheses. H1: The perceived authenticity of attitudes is greater for prejudice-prescribed groups than for prejudice-proscribed groups. H2: Perceived authenticity differs for fast versus slow responses and self versus others: For the self, slow responses should be judged more authentic (as slow responses should be more in line with participants’ desired responses), whereas when thinking of others’ attitudes, response desirability is not a relevant factor, and therefore fast and slow responses should be deemed equally (in)authentic (or fast responses might even be deemed more authentic). H3: Attitudinal conflict (i.e., the absolute difference between fast and slow responses) is greater for prejudice-proscribed (fast < slow) than prejudice-prescribed groups (fast = slow). H4: Greater attitudinal conflict leads to lower perceived authenticity, such that, if participants realize that they changed responses across trials, they might perceive lower authenticity in their responses.

On a methodological note, this research also hopes to contribute by adapting the two-response paradigm (which is widely used in research on judgment and metacognition; e.g., Bago et al., 2021; Thompson et al., 2011; Vaz and Mata, 2022; Vega et al., 2021) to capture automatic and controlled attitudinal responses toward social groups, thus bridging social cognition and other areas of cognition. Moreover, this paradigm is ideal for testing our hypotheses, as it (a) provides directly comparable measures of automatic and controlled responses and (b) enables an easy interpretation of those measures by laypeople (as compared to how they might interpret the results of, e.g., the IAT, priming, or affect misattribution).

## Method

### Participants and design

Two hundred sixteen English-speaking participants (57.1% female, 42.4% male, 0.5% Agender, *M*_age_ = 39.09, *SD*_age_ = 14.21) were recruited through the online participant recruitment platform *Prolific* to participate in a study on “Attitudes about social groups.” The sample size was determined in G*Power 3.1.9.7 (Faul et al., 2007) as that required to detect a small effect (*f* = 0.1) with *α* = 0.05 and Power = 0.95, in a mixed ANOVA with the design described (2 × 2 “measurements” within-subjects X 2 “groups” between-subjects)^1^.

The design was a 2 (trial: fast versus slow) X 2 (group type: prejudice-prescribed versus prejudice-proscribed) X 2 (source: self versus others), with the first two factors manipulated within-subjects and the third between-subjects.

### Materials

Participants reported their attitudes toward 10 groups. Five of the groups were prejudice-prescribed (i.e., groups against whom prejudice is warranted): rapists, terrorists, nazis, child molesters, and wife beaters; and five were prejudice-proscribed (i.e., groups against whom prejudice is frowned upon): Black people, people with AIDS, fat people, homeless people, and gays. The groups were adapted from previous research on the norms of prejudice expression (adapted from Crandall et al., 2002). Data from a pilot study confirmed that people find it more acceptable to express dislike toward the prejudice-prescribed groups (*M* = 8.38, *SD* = 1.56) than toward the prejudice-proscribed groups (*M* = 1.71, *SD* = 1.38; on a 9-point scale), *F*(1, 29) = 478.65, *p* < .001.

### Procedure

Participants gave their informed consent, provided demographic information (age and gender), and then began by reporting their attitudes toward each of the 10 groups. For each group, the name of the group appeared on screen, and participants were prompted to report their attitudes twice. First, participants were asked to report their attitude as fast as possible (*fast response*): “In the next step, you will see a word in the center of the screen. Please indicate, as fast as possible, what your attitude is towards what the word represents, by using the number keys in your keyboard (from 1 = extremely negative attitude to 9 = extremely positive attitude). It is of utmost importance that you give the first answer that comes to mind.” Participants then completed a response check, which asked “Is the answer that you just gave the first one that came to your mind?” (no/yes). Finally, participants were again asked for their attitude, but this time taking as long as they needed (*slow response*): “Please, state again your attitude towards the following group. This time you have no time pressure. Take all the time you need to think of your answer.” Participants started the task with a practice block where they indicated their attitudes toward “Lawyers” and then repeated the procedure for each of the 10 groups in randomized order.

After completing the two-response attitude-report task, participants again considered the 10 groups. This time, participants saw the fast and slow responses that they had previously given to each group and judged, for each response and each group, how authentic those responses were. For half of the participants, however, their previous responses were presented as “the most common answers previous participants gave,” so that they ostensibly judged the authenticity of other participants’ responses, not theirs. This procedure has been used in other research to ensure that any differences in judgment result from the (ostensible) responder and not from actual differences in responses (Alicke et al., 2001; Bell et al., 2019).

More specifically, participants went through one group at a time, in random order, and rated the authenticity of the fast response, followed by that of the slow response. For each response, authenticity was measured by two questions (on a 9-point scale: 1 - not at all, 5 - somewhat, and 9 - very much):

*When asked to express* [as fast as possible/without time constraints] [your/their] *attitude towards* [group], [your answer/the most common answer previous participants gave] *was* [response] (*on a scale from 1 – very negative to 9 – very positive*).

*How predictive is* [your/their] [fast slow] *response of* [your/their] *judgment and behavior towards* [group]*? That is, to what extent do you think that* [your/other people’s] *judgments and behaviors toward members of this group are guided by* [your/their] [initial gut/final pondered] *reaction* (*expressed in* [your/their] [fast/slow] *response*).

*To what extent does* [your/their] [fast/slow] *response reflect* [your/their] *true attitude towards* [group]*? That is, to what extent does* [your/other people’s] [initial gut/final pondered] *reaction* (*expressed in* [your/their] [fast/slow] *response*) *reflect how* [you/they] *really feel about members of this group.*

As the two measures were significantly correlated (*r* = 0.77), we standardized and aggregated them into a single authenticity composite.

## Results

### Attitudes

We started by excluding those trials for which participants reported not having given the first response that came to mind (2.5% of trials), which is standard procedure in this paradigm (e.g., Thompson et al., 2011; Vega et al., 2021). We entered the reported attitudes into a mixed model with trial (−0.5 = fast; +0.5 = slow) and group type (−0.5 = prejudice-prescribed; +0.5 = prejudice-proscribed) as fixed effects predictors, as well as their interaction. We included in the model the random effects of the subject and group to account for the non-independence of the responses. There was a main effect of trial, *b* = 0.12, *SE* = 0.04, *t*(3986.75) = 3.26, *p* = .001, 95% CI [0.05, 0.19], with slow responses being more positive (*M* = 3.64, *SD* = 2.88) than fast responses (*M* = 3.52, *SD* = 2.80). There was also a main effect of group type, *b* = 4.90, *SE* = 0.38, *t*(8.00) = 13.05, *p* < .001, 95% CI [4.04, 5.77], such that attitudes were more positive for prejudice-proscribed (*M* = 6.06, *SD* = 1.94) versus prejudice-prescribed groups (*M* = 1.15, *SD* = 0.62). More importantly, there was a trial-by-group type interaction, *F*(1, 3986.75) = 16.15, *p* < .001. Supporting H3 (*attitudinal conflict is greater for prejudice-proscribed than prejudice-prescribed groups*), slow responses were more positive than fast responses for prejudice-proscribed groups, *b* = 0.27, *SE* = 0.05, *t*(3986.75) = 5.12, *p* < .001, 95% CI [0.17, 0.37], but not for prejudice-prescribed groups, *b* = −0.03, *SE* = 0.05, *t*(3986.75) = −0.54, *p* = .589, 95% CI [−0.13, 0.07] (see Table 1).

**Table 1:** Average (SD) attitudes per group and trial.

Trial	Group type	
	Prejudice-proscribed	Prejudice-prescribed
Fast	5.92 (2.00)	1.16 (0.66)
Slow	6.19 (1.87)	1.13 (0.58)

### Authenticity

The composite score was entered into a mixed model that included trial (−0.5 = fast; +0.5 = slow), group type (−0.5 = prejudice-prescribed; +0.5 = prejudice-proscribed), source (−0.5 = others; +0.5 = self), and all interaction terms. We also included random effects of the subject and group to account for response non-independence. Consistent with H1, we found a main effect of group type, such that attitudes toward prejudice-prescribed groups were perceived as more authentic than for prejudice-proscribed groups, *b* = −0.51, *SE* = 0.06, *t*(7.98) = −8.85, *p* < .001, 95% CI [−0.64, −0.37]. There was a main effect of trial, *b* = 0.09, *SE* = 0.02, *t*(3983.18) = 4.02, *p* < .001, 95% CI [0.04, 0.13], with slow responses judged as more authentic. This was qualified by an interaction with group type, *F*(1, 3983.18) = 44.65, *p* < .001: Although slow responses were judged as more authentic than fast responses for prejudice-proscribed groups, *b* = 0.23, *SE* = 0.03, *t*(3983.18) = 7.53, *p* < .001, 95% CI [0.17, 0.29], this was not the case for prejudice-prescribed groups, *b* = −0.06, *SE* = 0.03, *t*(3983.18) = −1.89, *p* = .059, 95% CI [−0.12, 0.00]. There were no overall source differences, *b* = 0.05, *SE* = 0.08, *t*(213.14) = 0.61, *p* = .546, 95% CI [−0.11, 0.21], but there was a trial-by-source interaction that supports H2, *F*(1, 3983.18) = 14.37, *p* < .001: When considering one’s attitudes, slow responses were considered more authentic than fast ones, *b* = 0.17, *SE* = 0.03, *t*(3983.18) = 5.54, *p* < .001, 95% CI [0.11, 0.23], but for others no significant differences emerged, *b* = 0.005, *SE* = 0.03, *t*(3983.18) = 0.16, *p* = .872, 95% CI [−0.05, 0.06] (see Table 2). Finally, neither the group type-by-source, *F*(1, 1984.90) = 2.49, *p* = .115, nor the three-way interaction were significant, *F*(1, 3983.18) = 0.12, *p* = .727.

**Table 2:** Average (SD) authenticity per group, trial, and source.

Source	Prejudice-proscribed		Prejudice-prescribed	
	Fast	Slow	Fast	Slow
Self	−0.37 (0.99)	−0.06 (0.90)	0.24 (0.96)	0.28 (0.95)
Others	−0.38 (0.86)	−0.22 (0.84)	0.32 (0.77)	0.17 (0.89)

### Conflict

We calculated conflict by computing the absolute difference between fast and slow responses. Attitudinal conflict was higher for prejudice-proscribed (*M* = 0.60, *SD* = 0.86) versus prejudice-prescribed groups (*M* = 0.13, *SD* = 0.55), *t*(8.02) = 11.45, *p* < .001, as predicted by H3.

We tested H4 by entering conflict (and all interactions) into the model described in the previous subsection. There was a main effect of conflict, *b* = −0.22, *SE* = 0.02, *t*(4120.87) = −12.54, *p* < .001, 95% CI [−0.25, −0.18], such that, as predicted in H4, the greater the difference between fast and slow responses, the less participants regarded responses as authentic (see Supplementary Table 1, in OSF, for full results)^2^.

## Discussion

The present study assessed people’s perceptions of the authenticity of attitudes toward social groups. More specifically, it tested differences in perceived authenticity in the attitudes reported by self and other people. Results support the hypothesis that, as negative automatic attitudes may be inconsistent with one’s self-concept (particularly toward prejudice-proscribed groups), controlled attitudes expressed by the self, which tend to be more positive than automatic attitudes, are perceived as more authentic. When judging others, however, automatic and controlled responses were judged as equally authentic. Presumably, as others’ prejudice is not a threat, people feel no need to discount their automatic attitudes. Similarly, the greater perceived authenticity of slow responses was circumscribed to prejudice-proscribed groups. For prejudice-prescribed groups, as prejudice is not socially undesirable, people were less skeptical of reported attitudes.

Regarding the attitudinal responses themselves, our results are fully congruent with Devine’s (1989) findings on how low-prejudice responses occur from the inhibition of prejudiced attitudes. Rather than considering individual differences in the magnitude of prejudice, we manipulated instead social constraints on the expression of prejudice. We found that for prejudice-proscribed target groups, people have negative automatic response tendencies that are then suppressed in their controlled responses. These results add to previous research by demonstrating this attitudinal conflict with a different methodology.

Our study also shows that this attitudinal conflict predicts perceptions of authenticity: The greater the difference between automatic and controlled responses, the less authentic responses were considered. Overall (though with some variations depending on whether responses were automatic or controlled and targeted at prejudice-prescribed or -proscribed groups^2^), this held for both self and others. Thus, being aware of their attitudinal conflict, people might not only judge their responses as less authentic but also assume that others are similarly conflicted and cast doubt on the authenticity of others’ responses (in line with research on social metacognition; Mata, 2019, 2020; Mata et al., 2013a, b; Simão and Mata, 2023).

Future research should further test the relationship between attitudinal conflict and perceived authenticity and how it varies across social sources using other methods. It is possible that participants recognized their responses even though they were presented as ostensibly others’ and, therefore, did not judge them as less authentic than when those responses were presented as theirs. Still, this potential methodological limitation only works against our predicted effect, and therefore, the fact that we observed self-other differences using this paradigm is, we believe, all the more impressive. The interesting aspect of using this method to study self-other differences is that participants in different conditions are judging the same responses, but they interpret them differently depending on whether they are ascribed to self or others (see also Alicke et al., 2001; Bell et al., 2019).

One may also speculate on the reasons for participants’ skepticism about the attitudes expressed by others (or at least the fact that they do not consider other people’s controlled attitudinal responses to be more authentic than others’ automatic responses). This might mean that people are skeptical either of other people’s positive attitudes (or rather, their ability to suppress negative automatic response tendencies) or of others’ ability to express those attitudes authentically (i.e., skeptical that other people have developed a mature self-concept that enables them to understand their attitudes and values; Liesenfeld et al., 2024). Both these (attitudinal response control and authentic expression) require effortful deliberative thinking and maturation of key cognitive and metacognitive (self-regulatory and self-reflective) skills (Devine, 1989; Govorun and Payne, 2006; Liesenfeld et al., 2024), which participants might believe that other people lack in comparison to them (Garrison et al., 2022; Mata, 2024; Mata et al., 2013a, b). Future research should elucidate exactly the skills that people believe others lack, which make them doubt others’ authenticity when they express positive attitudes toward social groups.

We believe this research has implications for social cognition and interpersonal relations. Because sharing attitudes with others is essential to positive social interactions (Zorn et al., 2022), it becomes important to consider the authenticity of those attitudes. Perceived authenticity can have consequences for interpersonal relationship satisfaction in several ways. People feel more satisfied with their relationships and social interactions the more they perceive them as authentic (Rivera et al., 2019). Moreover, if others’ attitudes are perceived as inauthentic, attitudinal similarity between self and others will not be regarded as real. Furthermore, insofar as collectives are seen as less authentic in general, this may serve as a perpetuating mechanism of people’s cynicism toward society.

## Data Availability

The datasets presented in this study can be found in online repositories. The names of the repository/repositories and accession number(s) can be found at: https://osf.io/sfw6n/.

## Footnotes

^1^We report results from linear mixed models, as they account for possible effects of participant and group. However, the ANOVA described here yielded the same results (see this analysis in the OSF supplementary files).

^2^There was also a four-way interaction, *F*(1, 3975.20) = 8.49, *p* = .004, so we decomposed the analyses across prejudice-prescribed and prejudice-proscribed groups. Prejudice-prescribed groups. There was a main effect of conflict: The more participants changed responses from fast to slow trials, the less they perceived responses to be authentic, *b* = −0.24, SE = 0.03, *t*(4093.17) = −8.15, *p* < .001, 95% CI [−0.30, −0.18]. This effect was qualified by source and trial, *F*(1, 3975.20) = 25.31, *p* < .001. Whereas, for the self, conflict only affected the perceived authenticity of fast responses, *b* = −0.59, SE = 0.05, *t*(4068.34) = −11.69, *p* < .001, 95% CI [−0.69, −0.49] (slow responses, *b* = −0.05, SE = 0.05, *t*(4068.34) = −0.95, *p* = .344, 95% CI [−0.15, 0.05]), for others, it affected the perceived authenticity of fast and slow responses equally: conflict, *b* = −0.16, SE = 0.05, *t*(4063.53) = −3.60, *p* < .001, 95% CI [−0.25, −0.07]. conflict-by-trial, *F*(1, 3975.20) = 0.002, *p* = .962. Prejudice-proscribed groups. There was a main effect of conflict: The more different fast and slow responses were, the less authentic they were considered, *b* = −0.19, SE = 0.02, *t*(4117.08) = −11.89, *p* < .001, 95% CI [−0.22, −0.16]. There was also a conflict-by-source-by-trial interaction, *F*(1, 3975.20) = 10.80, *p* = .001. For self-responses, there was an effect of conflict for both fast responses, *b* = −0.45, SE = 0.03, *t*(4062.44) = −14.26, *p* < .001, 95% CI [−0.51, −0.39], and slow responses, *b* = −0.8, SE = 0.03, *t*(4062.44) = −2.44, *p* = .015, 95% CI [−0.14, −0.02]. Conversely, for others, there was only an effect of conflict for fast responses, *b* = −0.21, SE = 0.03, *t*(4061.48) = −7.35, *p* < .001, 95% CI [−0.27, −0.16] (for slow responses, *b* = −0.03, SE = 0.03, *t*(4061.48) = −0.88, *p* = .378, 95% CI [−0.08, 0.03]).
